# Journal of Hydrotherapeutics

**Published:** 1887-06

**Authors:** 


*Journal of Hydrotherapeutics*. No. 1. April, 1887. London: The Scientific Publishing Company, Limited.

We are glad to welcome this new Journal, on a subject of steadily increasing interest. The practice of flushing the tissues for the purpose of washing out excreting matter has a good foundation in physiological experiment: the well-known effect of injection of fluids in restoring the contractility of exhausted muscle, the utility of massage in improving the nutrition of the tissues, and then the every-day observations of the bath-physician, are all in favour of an extension of the various methods of treatment by mineral waters.

The mere introduction of large quantities of water into the organism must be considered to be a potent therapeutic agency; and when we remember that the natural water is usually a thermal one, and always a saline, we have the three factors: fluid, warmth, and mineral drugs, all working simultaneously and harmoniously together.

The curative value of a course of the "waters" is becoming more and more recognised; but "even a slight acquaintance with the mineral waters of the Continent must suffice to convince the student of medicine that it would be as wise to let his patient help himself indiscriminately to the bottles in a dispensary, as to encourage a hap-hazard employment of solutions of minerals prepared in the laboratory of Nature."

Much true scientific work may be carried on at health resorts; and it is for the purpose of promoting progress in the scientific study of Medical Hydrology and Climatology that our new contemporary has been established. It cannot fail, at the same time, to be of much value to the patients who find it necessary to visit these resorts, and to the medical men who have to aid in the selection of a suitable one for the individual case.

